# Risk factors for poor cognition among older adults in low‐ and middle‐income countries

**DOI:** 10.1002/alz70860_100259

**Published:** 2025-12-23

**Authors:** Ashleigh S. Vella, Keshuo Lin, Darren M. Lipnicki, Blossom CM Stephan, Anbupalam Thalamuthu, Juan J. Llibre‐Rodriguez, Maelenn Guerchet, Pierre‐Marie Preux, Suzana Shahar, Ding Ding, Yuda Turana, Erico Costa, Shifu Xiao, Richard Walker, Stella‐Maria Paddick, Hugh C Hendrie, Sujuan Gao, Murali Krishna, Marcia Scazufca, Eleonora D'Orsi, Ricardo Oliveira Guerra, Oye Gureje, Vincent Mubangizi, Perminder S. Sachdev, Jiyang Jiang, COSMIC authors

**Affiliations:** ^1^ Centre for Healthy Brain Ageing (CHeBA), UNSW Sydney, Sydney, NSW, Australia; ^2^ Curtin University, Perth, Western Australia, Australia; ^3^ Dementia Research Unit. Medical University of Havana, Havana, Havana, Cuba; ^4^ National Institute for Health and Medical Research U1094, Institut de Recherche pour le Developpement UMR270, Epidemiology of Chronic Diseases in Tropical Zone, Institute of Epidemiology and Tropical Neurology, OmegaHealth, University Limoges, Centre Hosp, Limoges, France; ^5^ Laboratory of Chronic Diseases Epidemiology (LEMACEN), Faculty of Health Sciences, School of Health Sciences, University of Abomey‐Calavi (UAC), Cotonou, Benin; ^6^ Centre for Healthy Aging and Wellness, Faculty of Health Sciences, Universiti Kebangsaan Malaysia, Kuala Lumpur, Malaysia; ^7^ Institute of Neurology, Huashan Hospital, Fudan University, Shanghai, China; ^8^ Dept of Neurology, School of Medicine and Health Sciences, Atma Jaya Catholic University of Indonesia, Jakarta, Indonesia; ^9^ Instituto René Rachou da Fundaçaõ Oswaldo Cruz, Rio de Janeiro, Brazil; ^10^ Shanghai Mental Health Center, Shanghai Jiao Tong University School of Medicine, Shanghai, China; ^11^ Department of Medicine, North Tyneside General Hospital, Newcastle, Newcastle Upon Tyne, United Kingdom; ^12^ Institute of Neuroscience, Newcastle University, Newcastle, Newcastle Upon Tyne, United Kingdom; ^13^ Indiana University School of Medicine, Indianapolis, IN, USA; ^14^ School of Medicine, Indiana University, Indianapolis, IN, USA; ^15^ School of Medicine, Indiana University‐Purdue University at Indianapolis, Indianapolis, IN, USA; ^16^ Institute of Public Health, Bangalore, India; ^17^ Hospital das Clínicas, Faculdade de Medicina da Universidade de São Paulo, São Paulo, Brazil; ^18^ Federal University of Santa Catarina, Florianopolis, Brazil; ^19^ Department of Physical Therapy, Federal University of Rio Grande do Norte, Rio Grande do Norte, Brazil; ^20^ WHO Collaborating Centre for Research and Training in Mental Health, Neurosciences and Substance Abuse, Department of Psychiatry, University of Ibadan, Ibadan, Nigeria; ^21^ Family Medicine and Community Practice, Mbarara university of Science and Technology, Mbarara, Uganda

## Abstract

**Background:**

Research into an effective dementia treatment is ongoing. Therefore, identifying individuals at risk of declining cognition and dementia is fundamental for initiating modifiable risk factor interventions that can delay dementia onset. Research into modifiable risk factors has almost exclusively been from high‐income countries, despite 60% of individuals with dementia living in low‐ and middle‐income countries (LMICs). Addressing this research inequality, the current study examines cross‐sectional relationships between risk factors and cognitive performance in LMICs, with the aim of identifying modifiable risk factors particularly suitable for interventions in these regions.

**Method:**

Data were obtained from 15 members of the Cohort Studies of Memory in an International Consortium (COSMIC), representing 11 countries (Brazil, China, Colombia, Cuba, India, Indonesia, Malaysia, Nigeria, Republic of Congo, Tanzania, & Uganda) across 6 continents (participants: 53,136; *M_age_* = 70.75, *SD_age_* = 7.87; 57% female). We investigated (after harmonisation) the following risk factors: age, APOE ε4, anxiety, body mass index (BMI), blood pressure, cardiovascular disease, cholesterol, depression, diabetes, education, excessive alcohol, hypertension, hearing loss, physical activity, sex, smoking status, and stroke history. Harmonised global cognition was the outcome. On the AD Workbench, we performed linear regressions for each risk factor within each study at baseline. Cross‐sectional results from all studies were pooled in a multivariate meta‐analysis, with a random intercept for Country and study. We investigated age, sex and education as risk factors, and included them as covariates when analysing other factors.

**Result:**

Older age (β=**‐**.231, *p* < .001), being male (β=.151, *p* < .001), less education (β=1.569, *p* < .001), history of angina (β=**‐**.099, *p* < .001), anxiety (β=**‐**.262, *p* < .001), depression (β=**‐**.203, *p* < .001), stroke (β=**‐**.375, *p* < .001), diabetes (β=**‐**.060, *p* = .002), excessive alcohol consumption (β=**‐**.190, *p* < .001), currently smoking (β=**‐**.097, *p* < .001), and less physical activity (β=.160, *p* < .001) were significantly associated with worse global cognition. Heterogeneity in the relationship between risk factors and cognition exists across countries, warranting further analyses for each LMIC.

**Conclusion:**

Findings suggest that risk factor interventions may help to reduce dementia in LMICs, though country level heterogeneity in the effects suggests that tailoring interventions to regions will be needed. Future research will explore how the factors we identified predict incidence of dementia in LMICs using Machine Learning models.

